# Comparative effects of exercise modalities and dose parameters on chronic low back pain in adults: a systematic review and network meta-analysis

**DOI:** 10.3389/fphys.2026.1836061

**Published:** 2026-05-14

**Authors:** Li Wang, Chengyu Zhou, Cuili Zhai

**Affiliations:** 1 Department of Physical Education, Anhui Sanlian University, Anhui, China; 2 Strength and Conditioning Training College, Beijing Sports University, Beijing, China; 3 School of Chinese Martial Arts, Beijing Sport University, Beijing, China

**Keywords:** chronic low back pain, dose-response, exercise therapy, network meta-analysis, rehabilitation

## Abstract

**Objective:**

Although exercise therapy is widely recommended for chronic low back pain (CLBP), the comparative effectiveness of specific exercise modalities and optimal dose parameters remains unclear. This study aimed to evaluate and rank different exercise types and key dose-related factors using a network meta-analysis.

**Methods:**

A systematic search of PubMed, Web of Science, Embase, Cochrane Library, and CNKI was conducted from database inception to December 2025. Randomised controlled trials involving adults with non-specific CLBP were included. Network meta-analysis was performed using Review Manager 5.4 and Stata 18.0. The relative efficacy of interventions was ranked using surface under the cumulative ranking curve (SUCRA).

**Results:**

A total of 24 randomised controlled trials were included, involving 28 intervention arms. All exercise modalities (stabilisation, resistance, traditional, combined, and motor control-based approaches) demonstrated significant improvements in pain compared with control conditions (p < 0.05). Exercise sessions lasting 15–20 min, 30–40 min, and 45–50 min were all effective, with no significant differences between durations. Frequencies of 1–2, 3, and 6–7 sessions per week significantly reduced pain, with 6–7 sessions per week ranking highest. Intervention periods of ≥16 weeks showed the greatest efficacy. SUCRA rankings indicated that traditional exercise (SUCRA = 87.9), 15–20 min per session (SUCRA = 91.6), 6–7 sessions per week (SUCRA = 91.5), and ≥16 weeks duration (SUCRA = 97.9) were most likely to be optimal.

**Conclusion:**

Exercise therapy is effective for managing CLBP in adults. The most favourable combination may represent traditional exercise modalities performed for 15–20 minutes per session, 6–7 times per week, over at least 16 weeks. These findings provide evidence to inform more precise exercise prescription for CLBP rehabilitation.

**Systematic review registration:**

https://www.crd.york.ac.uk/prospero/, identifier.

## Introduction

CLBP is defined as persistent or recurrent pain in the lumbar region lasting more than 12 weeks. Its aetiology is typically complex and non-specific, and the condition is frequently associated with functional limitations and psychological distress (Taheri et al., 2025). As a major global public health challenge, CLBP accounts for 8.1% of all disability-adjusted life years worldwide and remains the leading cause of disability across diseases (An et al., 2025). Among adults, the annual incidence reaches 38.9%, with a lifetime prevalence exceeding 60% (GBD, L.B.P.C., 2023). This high disease burden severely compromises physical function and quality of life, reduces work productivity, and imposes substantial economic costs on healthcare systems (Patel and Patel, 2021). In Europe and North America alone, the annual direct and indirect costs attributable to CLBP exceed one trillion dollars, underscoring the urgent need to address this pervasive public health issue (Saki and Ziya, 2025).

Current clinical guidelines endorse treatment strategies centred on non-pharmacological interventions to alleviate pain, restore spinal function, and lower recurrence risk (Šajnović et al., 2024; Zang and Yan, 2024). However, while oral non-steroidal anti-inflammatory drugs offer short term relief, prolonged use carries risks of adverse reactions, including gastrointestinal and cardiovascular complications (Wehling, 2014). Furthermore, physical therapies like traction and massage demonstrate limited long term efficacy, with patient recurrence rates remaining as high as 60%-80% within one year (Harte et al., 2003). Consequently, developing safe, effective, and sustainable non-pharmacological interventions is critical to overcoming current therapeutic limitations for CLBP. Exercise therapy, as a core non-pharmacological approach, is strongly supported by evidence-based research for management (Xu et al., 2023). Pathophysiologically, exercise mitigates CLBP through multiple mechanisms. First, it strengthens core muscles and improves coordination, thereby enhancing spinal stability and reducing abnormal loading on intervertebral discs and nerve roots (Zou and Hao, 2024). Second, it modulates pain pathways, inhibits central sensitization, increases endorphin secretion, and elevates pain thresholds. Additionally, exercise improves spinal flexibility and joint range of motion, which can alleviate muscle spasms and localized inflammation (Song et al., 2024). Given these mechanistic benefits, combined with its safety, low cost, and practicality—exercise therapy has been widely recommended in recent clinical studies and remains a focal point of CLBP intervention research (Rainville et al., 2004; Zang and Yan, 2024).

Previous research has demonstrated the overall benefits of exercise intervention compared to conventional care or pharmacotherapy. For instance, Hayden et al. found that mat-based exercises and McKenzie therapy provided significantly greater short-term pain relief and functional improvement than control conditions (Hayden et al., 2021). Furthermore, IJzelenberg et al. confirmed that exercise intervention could reduce the three-year recurrence rate of CLBP by approximately 30% (IJzelenberg et al., 2023). However, these studies have typically been limited to direct pairwise comparisons of interventions. As such, most previous studies have focused on pairwise comparisons or synthesizing indirect evidence only for exercise modalities, rather than comprehensively integrating both exercise types and critical dose−response parameters (session duration, frequency, intervention period) within a unified network meta−analysis (Li et al., 2023). Moreover, prior research has largely focused on the aggregate effect of exercise, lacking a detailed dose-response analysis of its core components (e.g., type, duration, frequency). This gap has hindered the translation of evidence into clear, optimal exercise prescriptions for clinical practice.

Accordingly, this study utilized network meta-analysis to systematically review and analyse randomized controlled trials comparing exercise interventions for adults with CLBP. We evaluated the relative efficacy of different exercise types for pain relief and functional recovery and analysed the dose-response effects of key parameters, including time, frequency, and period. The goal is to provide high quality evidence to support the clinical development of individualized, precise exercise prescriptions for CLBP and to help alleviate the public health burden.

## Methods

### Search strategy

Search PubMed, Web of Science, Embase, Cochrane, and CNKI. The search period covers from the establishment of each database to December 2025. The full search strategy was constructed using Boolean operators (AND/OR/NOT) and controlled vocabulary (MeSH/Emtree) for three core components: (1) “exercise” OR “strength training” OR “weight Training” OR “physical exercise” OR “physical activity” OR “sports” OR “fitness” OR “cardio training” OR “exercise therapy” OR “taichi” OR “qigong” OR “baduanjin “OR”wuqinxi”; (2) “adult” OR “grown-up” OR “adulthood” OR “middle-aged” OR “elderly” OR “senior” OR “professional” OR “independent” OR “mature in-dividual”OR”established person”; (3)”CLBP” OR “chronicback pain” OR “lumbar pain” OR “low backache” OR “chroniclumbago” OR “persistent backache” OR “chronic lumbar dysfunction” OR “persistent lumbosacral pain” OR “musculoskeletal pain” OR “disc degeneration” OR “sacroiliacjoint dysfunction”. To ensure methodological rigor and transparency, the study protocol was registered and approved on the PROSPERO international prospective register of systematic reviews prior to commencement (Registration number: CRD420261283047).

### Inclusion and exclusion criteria

Studies were included if they met the following criteria: (1) Population: adults aged ≥18 years with non-specific chronic low back pain (CLBP) lasting ≥12 weeks, excluding CLBP caused by osteoporosis, fracture, inflammatory lesions, or other specific pathologies; (2) Comparators: experimental group received targeted exercise therapy (stabilisation, resistance, traditional, combined, motor control-based exercise); control group received no exercise, routine exercise (ROE), routine care, or passive physical therapy; (3) Primary outcomes for network meta-analysis: pain intensity measured by Visual Analogue Scale (VAS); (4) The study type was a randomized controlled trial.

Studies were excluded for any of the following: (1) Non-randomized controlled research design; (2) Literature types such as reviews, comments, without clear description and unable to contact the author for confirmation; (3) Studies with incomplete important data; (4) Low back pain involving specific pathologies such as osteoporosis, fractures, inflammatory lesions;

### Data extraction

Study selection was performed in duplicate and independently by two trained reviewers. First, duplicates were removed using EndNote X20. Second, titles and abstracts were screened against eligibility criteria. Third, full texts of potentially relevant records were assessed for final inclusion. Any disagreements were resolved by consensus or consultation with a third senior reviewer. A flow diagram of the selection process was prepared in accordance with PRISMA-NMA guidelines. Any discrepancies encountered during these processes were resolved through consultation with a third, senior member of the research team, whose extensive experience in adult chronic low back pain treatment informed the final consensus on study inclusion or exclusion. A standardized, pre-piloted data extraction form was used. Two reviewers independently extracted the following data: study characteristics (author, year, country), participant demographics (age, sample size, CLBP duration), intervention details (type, duration, frequency, period), comparator information, and outcome measures. Discrepancies were checked and resolved by cross-verification. All extracted data were double-checked to ensure accuracy.

### Statistical analysis

Network meta-analysis was performed using Review Manager 5.4 and Stata 18.0. A random−effects model was applied to account for potential clinical and methodological heterogeneity across trials. Network meta-analysis assumptions were strictly evaluated: (1) Transitivity: Assessed by comparing baseline characteristics (age, CLBP duration, baseline pain) across included trials to ensure similarity of effect modifiers; (2) Inconsistency: Evaluated using the node-splitting method to compare direct and indirect evidence; inconsistency was considered significant at p < 0.05. A consistency model was used if no significant inconsistency was detected. Since the outcome measure was pain intensity on the VAS, a continuous outcome, the SMD was selected as the effect size metric to enable standardized pooled effect estimation and consistent comparison across trials in the network meta-analysis. This ensured analytical consistency and comparability, allowing for an accurate pooled effect estimate. Network meta-analysis was employed to synthesize both direct and indirect evidence, enabling the simultaneous comparison of multiple interventions. In the generated network diagram, each node represents an intervention, with its size proportional to the total sample size. The connecting lines indicate direct comparisons, with their thickness corresponding to the number of studies informing that comparison. To further evaluate the relative efficacy of the interventions, SUCRA provides a hierarchy of all included interventions on a scale from 0% to 100%; a higher SUCRA value indicates a greater probability of that intervention being the most effective for improving CLBP.

## Results

### Studies retrieved

**Figure 1:**
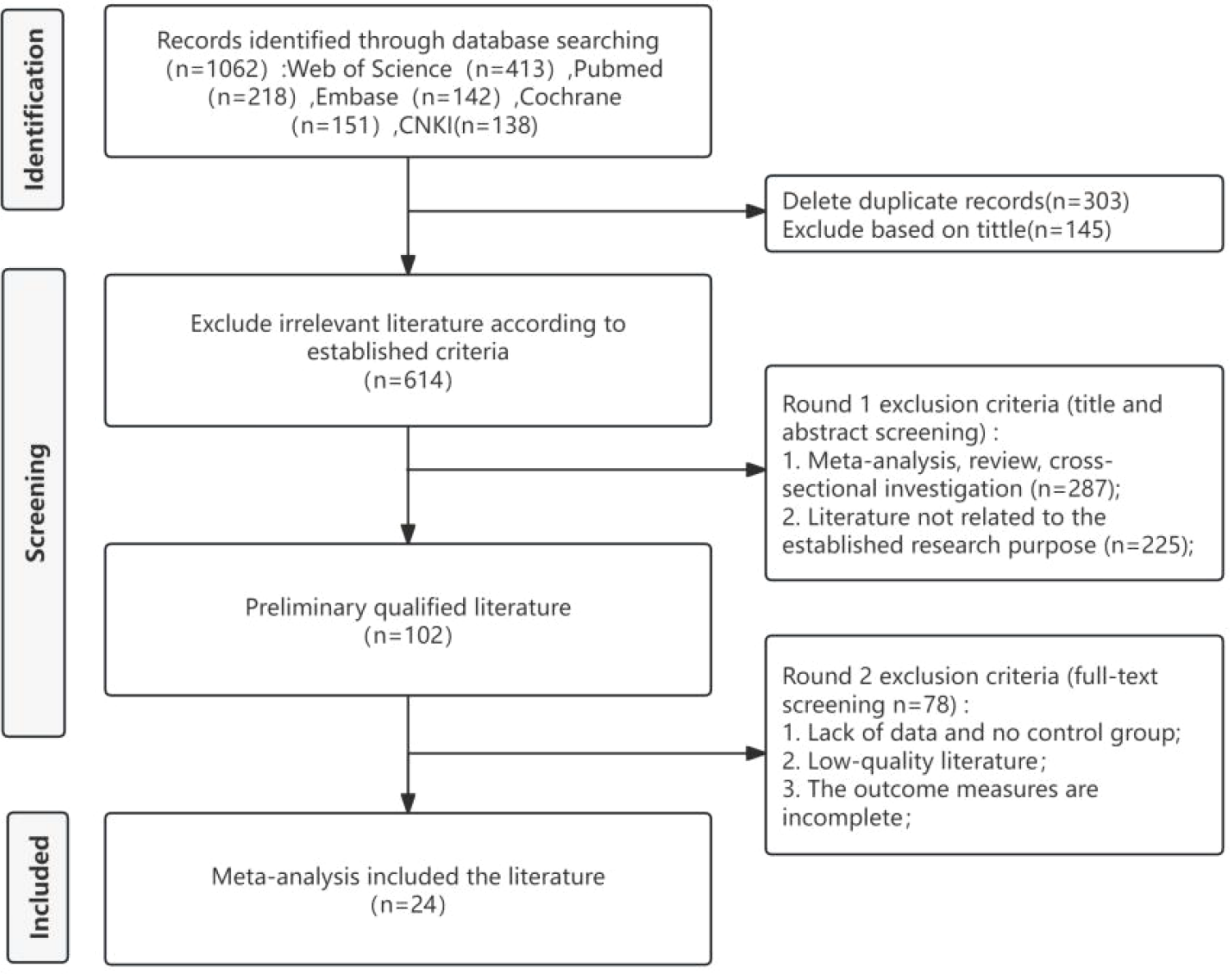
Flow chart of literature retrieval.

The initial database search retrieved 1,062 records. After removing 303 duplicates and 145 title records, 614 unique records underwent title and abstract screening, of which 512 were excluded. The remaining 102 articles were assessed for eligibility via full-text review based on the predefined inclusion and exclusion criteria, resulting in the final inclusion of 24 studies for the systematic review and meta-analysis. The complete literature selection process is illustrated in **Figure 1**.

### Research characteristics

The final sample comprised 24 studies, encompassing 28 distinct intervention groups. Among these, Lee et al. (2016) (Lee and Kang, 2016), Liu et al. (2019) (Liu et al., 2019), Oh et al. (2015) (Oh et al., 2015), and Teut et al. (2016) (Teut et al., 2016), each contributed two intervention arms. Exercise interventions in the experimental groups were categorized as SE, RE, MT, CE, or SLE. Control group interventions primarily involved ROE or RE. The intervention duration across studies ranged from 6 to 24 weeks, with a frequency of 1 to 7 sessions per week and individual session durations of 30 to 120 minutes. The basic characteristics of the included studies are presented in **Table 1**.

**Table 1:** Basic information table of included literatures.

Author	N (trial/con)	Age	Trial	Con	Time	Frequency	Period
Akhtar, 2017 (Akhtar et al., 2017)	53/55	45.50 ± 6.61	SE	ROE	60 min	2 times/week	6 weeks
Arampatzis, 2017 (Arampatzis et al., 2017)	20/20	31.4 ± 5.5	RE	ROE	90 min	2 times/week	13 weeks
Bae, 2018 (Bae et al., 2018)	18/18	32.4 ± 10.7	SE	ROE	30 min	3 times/week	12 weeks
Cho, 2014 (Cho et al., 2014)	15/15	44.0 ± 6.7	SE	ROE	40 min	3 times/week	6 weeks
Gladwell, 2006 (Gladwell et al., 2006)	20/14	45.9 ± 8.0	MT	ROE	60 min	1 times/week	6 weeks
Hwangbo, 2015 (Hwangbo et al., 2015)	15/15	34.0 ± 2.9	SE	ROE	50 min	3 times/week	6 weeks
Kumar, 2011 (Kumar, 2011)	9/9	22.5 ± 1.09	SE	ROE	15 min	Not reported	8 weeks
Lee, 2016 (Lee and Kang, 2016)	15/6	43.3 ± 9.9	CE	ROE	50 min	2 times/week	12 weeks
Lee, 2016 (Lee and Kang, 2016)	15/6	43.3 ± 9.9	SE	ROE	50 min	2 times/week	12 weeks
Liu, 2019 (Liu et al., 2019)	15/13	58.13 ± 5.38	TE	ROE	60 min	3 times/week	12 weeks
Liu, 2019 (Liu et al., 2019)	15/13	58.13 ± 5.38	SE	ROE	60 min	3 times/week	12 weeks
Michaelson, 2016 (Michaelson et al., 2016)	35/35	49.3 ± 14.0	SE	ROE	120 min	1 times/week	8 weeks
Pardis, 2018 (Noormohammadpour et al., 2018)	10/10	41.3 ± 6.4	SE	ROE	Not reported	Not reported	8 weeks
Oh, 2015 (Oh et al., 2015)	10/10	44.20 ± 2.70	MT	ROE	30 min	5 times/week	12 weeks
Oh, 2015 (Oh et al., 2015)	10/10	44.20 ± 2.70	SLE	ROE	30 min	5 times/week	12 weeks
Roh, 2016 (Roh et al., 2016)	53/49	50.5 ± 9.1	SLE	ROE	30 min	3 times/week	12 weeks
Shamsi, 2015 (Shamsi et al., 2015)	19/20	38.5 ± 11.9	SE	ROE	60 min	3 times/week	6 weeks
Tang, 2016 (Tang et al., 2016)	41/41	43.6 ± 6.4	SE	ROE	30 min	7 times/week	6 weeks
Tekur, 2012 (Tekur et al., 2012)	40/40	48.0 ± 4.0	MT	ROE	45 min	7 times/week	4 weeks
Teut, 2016 (Teut et al., 2016)	61/57	73.0 ± 5.6	MT	ROE	45 min	2 times/week	12 weeks
Teut, 2016 (Teut et al., 2016)	68/57	73.0 ± 5.6	TE	ROE	45 min	2 times/week	12 weeks
Ulger, 2017 (Ulger et al., 2017)	57/56	41.6 ± 12.9	SE	ROE	60 min	3 times/week	6 weeks
Ulger, 2023 (Ulger et al., 2023)	16/12	55.08 ± 2.67	MT	STE	60 min	2 times/week	8 weeks
Williams, 2005 (Williams et al., 2005)	16/17	48.00 ± 1.96	MT	ROE	30 min	5 times/week	16 weeks
Williams, 2009 (Williams et al., 2009)	43/47	48.40 ± 1.86	MT	ROE	30 min	7 times/week	24 weeks
Yoo, 2012 (Yoo and Lee, 2012)	15/15	20.5 ± 0.5	SLE	ROE	45 min	3 times/week	4 weeks
You,2014 (You et al., 2014)	20/20	51.30 ± 7.0	SE	ROE	40 min	3 times/week	8 weeks
Zhang,2015 (Zhang et al., 2015)	46/46	51.62 ± 4.03	SLE	STE	40 min	7 times/week	8 weeks

### Risk assessment

**Figure 2:**
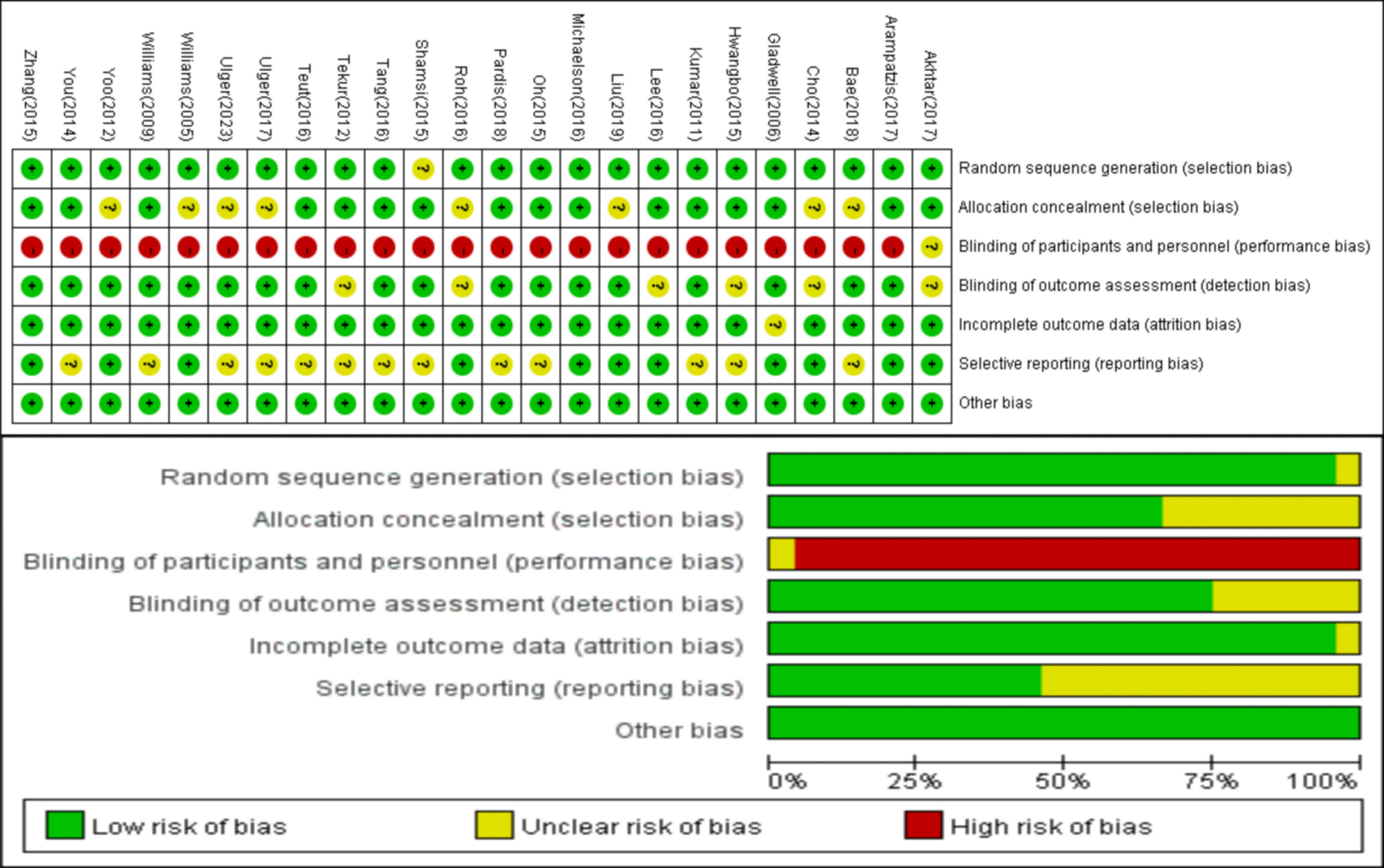
Risk of bias of the included studies.

The quality of the 24 included RCTs was assessed using the Cochrane Risk of Bias tool, and Review Manager 5.4. Detailed results of the literature quality evaluation are presented in **Figure 2**, the evaluation focused on the following aspects: (1) Selection bias: whether the method of random sequence generation was used; (2) Allocation concealment: whether the allocation was effectively concealed; (3) Blinding: whether the subjects or investigators were blinded; (4) Data integrity: whether missing data was adequately reported and intention-to-treat analysis was applied; (5) Selective reporting: whether there was any selective reporting of outcomes; (6) Other biases: whether other factors contributed to potential bias.

### Result of the network relationship

**Figure 3:**
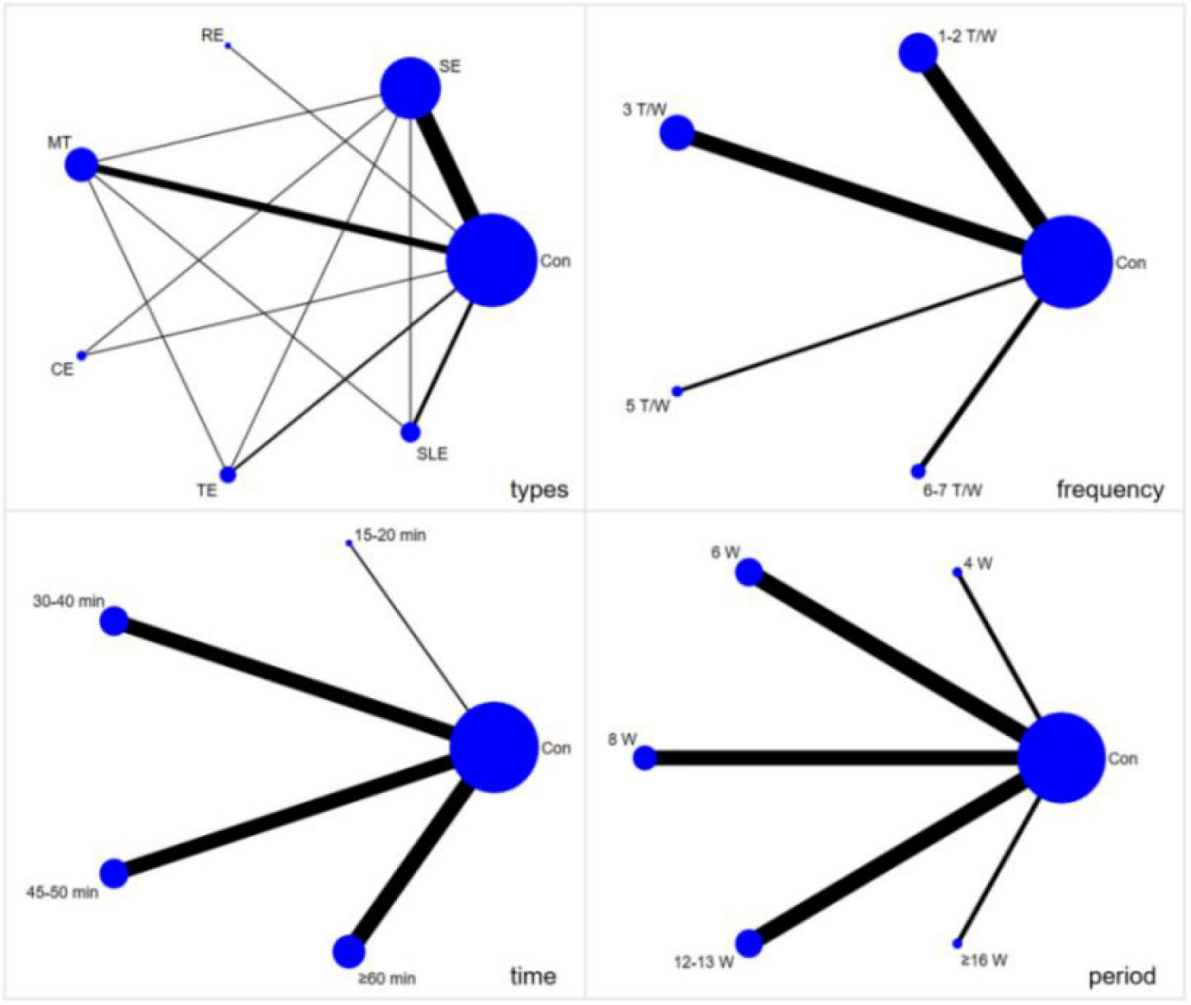
The network relationship diagram of different exercise factors.

The results of the network relationship are shown in **Figure 3**. For exercise types, the network included 7 nodes and 14 direct comparisons; no disconnected nodes were identified. The control group was directly compared with SE, MT, and SLE in the largest number of trials. For exercise frequency, the network included 5 nodes and 9 direct comparisons, with the most direct comparisons between control and 1–2 times/week or 3 times/week. For session duration, the network included 5 nodes and 8 direct comparisons, with the most direct comparisons between control and 30–40 min, 45–50 min, or ≥60 min. For intervention period, the network included 6 nodes and 11 direct comparisons, with the most direct comparisons between control and 6 weeks, 8 weeks, or 12–13 weeks. Line thickness corresponds to the number of studies informing each direct comparison.

### Result of different exercise factors

The comparative efficacy of different exercise types is presented in **Table 2**. The results indicate that, compared to the control group, SE, RE, MT, CE, and TE all significantly improved chronic low back pain in adults. The point estimates suggested that RE [SMD = −0.86, 95% CI (−3.43, −1.72)], MT [SMD = −0.93, 95% CI (−1.66, −0.20)], CE [SMD = −0.50, 95% CI (−2.90, −1.91)], and TE [SMD = −0.56, 95% CI (−2.08, −0.20)] were among the most effective interventions. Furthermore, the network comparisons revealed that TE was significantly more effective than SE, RE, MT, and CE (p < 0.05).

**Table 2:** League table of the influence of different exercise types on CLBP.

CON	SE	RE	MT	CE	TE	SLE
0.77 (-3.38,-1.84)	**SE**					
-0.86 (-3.43,-1.72)	0.43 (-2.08,2.95)	**RE**				
-0.93 (-1.66,-0.20)	-0.63 (-2.04,0.79)	-1.06 (-3.75,1.63)	**MT**			
-0.50 (-2.90,-1.91)	0.11 (-2.51,2.72)	-0.33 (-3.80,3.15)	0.73 (-2.05,3.52)	**CE**		
0.56 (-2.08,-0.20)	-1.76 (-4.37,-0.85)	-2.19 (-5.67,-1.28)	-1.13 (-3.92,-1.65)	-1.87 (-5.42, -1.68)	**TE**	
1.38 (-1.38, 4.13)	0.12 (-2.47,2.71)	-0.31 (-3.77,3.14)	0.75 (-2.02, 3.51)	0.01 (-3.52, 3.55)	1.88 (-1.65, 5.41)	**SLE**

The effects of exercise time are summarized in **Table 3**. The analysis indicated that interventions lasting 15-20min, 30-40min, and 45-50min all resulted in significantly greater improvement in chronic low back pain compared to the control group. There were no statistically significant differences in efficacy among these three duration groups. The standardized mean differences were as follows: 15–20 min [SMD = −2.66, 95% CI (−4.95, −0.37)], 30–40 min [SMD = −1.54, 95% CI (−2.29, −0.79)], 45–50 min [SMD = −0.89, 95% CI (−1.63, −0.14)]. However, no statistically significant differences in efficacy were observed among these categories (p > 0.05). In terms of SUCRA ranking, 15–20 min showed the highest probabilistic rank (SUCRA = 91.6), but this ranking reflects relative probability rather than statistically significant superiority over other durations.

**Table 3:** League table of the influence of different exercise time on CLBP.

CON	12-20min	30-40min	45-50min	≥60min
-2.66 (-4.95, -0.37)	**15–20 min**			
-1.54 (-2.29, -0.79)	1.12 (-1.29, 3.53)	**30–40 min**		
-0.89 (-1.63, -0.14)	1.78 (-0.63, 4.19)	0.66 (-0.40, 1.71)	**45–50 min**	
-0.65 (-1.34, 0.05)	2.01 (-0.38, 4.41)	0.89 (-0.13, 1.92)	0.24 (-0.78, 1.26)	**≥60 min**

The comparative effects of exercise frequency are presented in **Table 4**. The results indicate that intervention frequencies of 1–2 T/W, 3 T/W, and 6–7 T/W each yielded significantly greater improvement in chronic low back pain compared to the control condition. The corresponding effect sizes were: 1–2 times/week [SMD = −0.82, 95% CI (−1.50, −0.15)], 3 times/week [SMD = −1.06, 95% CI (−1.78, −0.33)], 6–7 times/week [SMD = −1.86, 95% CI (−3.04, −0.68)]. Furthermore, the 6–7 T/W frequency was found to be significantly more effective than all other frequency groups analysed (p < 0.05).

**Table 4:** League table of the influence of different exercise frequency on CLBP.

CON	1-2T/W	3T/W	5T/W	6-7T/W
-0.82 (-1.50, -0.15)	**1–2 T/W**			
-1.06 (-1.78, -0.33)	-0.24 (-1.23, 0.76)	**3 T/W**		
-0.33 (-1.80, 1.15)	0.50 (-1.13, 2.12)	0.73 (-0.91, 2.38)	**5 T/W**	
-1.86 (-3.04, -0.68)	-1.04 (-2.40, -0.32)	-0.80 (-2.19, -0.58)	-1.54 (-3.42, -0.35)	**6–7 T/W**

The effects of different exercise period are detailed in **Table 5**. The analysis demonstrated that programs lasting 8 weeks [SMD = −1.13, 95% CI (−1.96, −0.31)], 12–13 weeks [SMD = −1.02, 95% CI (−1.77, −0.27)], ≥16 weeks [SMD = −2.64, 95% CI (−4.06, −1.23)] resulted in significantly greater improvement in chronic low back pain compared to the control condition. The 4 weeks intervention showed a positive but non-significant trend (SMD = -0.81, 95% CI [-2.18, 0.56]). Furthermore, the ≥16 weeks intervention period was found to be significantly more effective than all other duration periods analysed (p < 0.05).

**Table 5:** League table of the influence of different exercise period on CLBP.

CON	4W	6W	8W	12-13W	≥6W
-0.81 (-2.18, 0.56)	**4 W**				
-0.69 (-1.42, 0.04)	0.12 (-1.43, 1.68)	**6 W**			
-1.13 (-1.96, -0.31)	-0.32 (-1.92, 1.27)	-0.45 (-1.54, 0.65)	**8 W**		
-1.02 (-1.77, -0.27)	-0.21 (-1.77, 1.35)	-0.33 (-1.38, 0.72)	0.12 (-1.00, 1.23)	**12–13 W**	
-2.64 (-4.06, -1.23)	-1.83 (-3.80, 0.13)	-1.95 (-3.54, -0.36)	-1.51 (-3.14, 0.12)	-1.62 (-3.22, -0.03)	**≥16 W**

**Figure 4:**
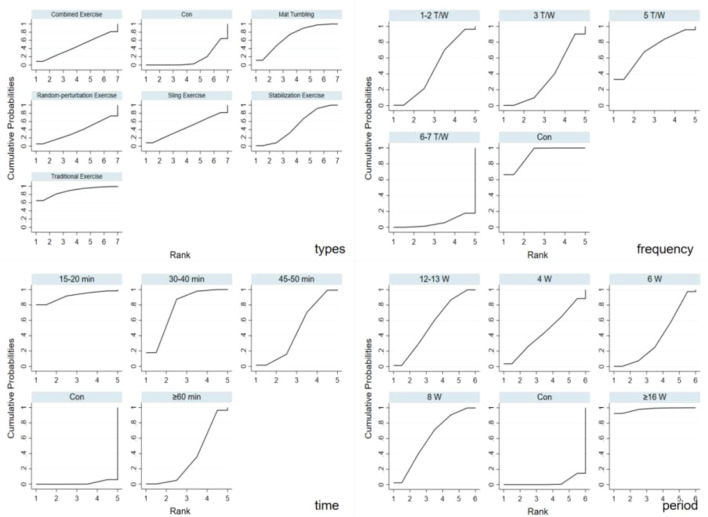
The SUCRA of the intervention effect on the dose of exercise factors.

The cumulative ranking of different exercise dosage elements on CLBP in adults is illustrated in **Figure 4**, with detailed SUCRA values provided in **Table 5**. SUCRA values reflect the relative probabilistic hierarchy of interventions rather than definitive clinical or statistical superiority, especially given overlapping confidence intervals among most comparisons. The ranking of SUCRA based on the exercise types is as follows: TE (SUCRA = 87.9) was ranked highest, followed by MT (SUCRA = 70.1), SE (SUCRA = 50.1), SLE (SUCRA = 45.3), CE(SUCRA = 5.1), RE(SUCRA = 37.0), and the CON (SUCRA = 14.7). In terms of time, the most favourable ranking was for 15–20 min (SUCRA = 91.6), followed by 30–40 min (SUCRA = 75.8), 40–50 min(SUCRA = 46.7), 60-min (SUCRA = 34.4), and the CON (SUCRA = 1.5). In terms of time frequency, an intervention frequency of 6–7 T/W was ranked highest (SUCRA = 91.5), followed by 5 T/W (SUCRA = 70.1), 1–2 T/W (SUCRA = 47.1), 3 T/W (SUCRA = 35.1), and the CON (SUCRA = 6.1). In terms of period, a program duration of ≥16 weeks was ranked highest (SUCRA = 97.9), followed by 8 weeks (SUCRA = 60.7), 12–13 weeks (SUCRA = 55.3), 4 weeks (SUCRA = 45.3), 6 weeks (SUCRA = 37.7), and the CON (SUCRA = 3.0). These rankings should be interpreted cautiously as they do not confirm clinically meaningful differences when confidence intervals overlap.

### Sensitivity analysis

This study adopted the method of elimination one by one for sensitivity analysis. The results indicated that sequentially excluding any single study did not substantially alter the direction or statistical significance of the pooled effect size, indicating that the findings are robust. Furthermore, although the exclusion of individual studies occasionally reduced the I² value, the overall impact on the heterogeneity estimate was minimal, confirming that no single study exerted a dominant influence on the analysis.

### Publication bias

**Figure 5:**
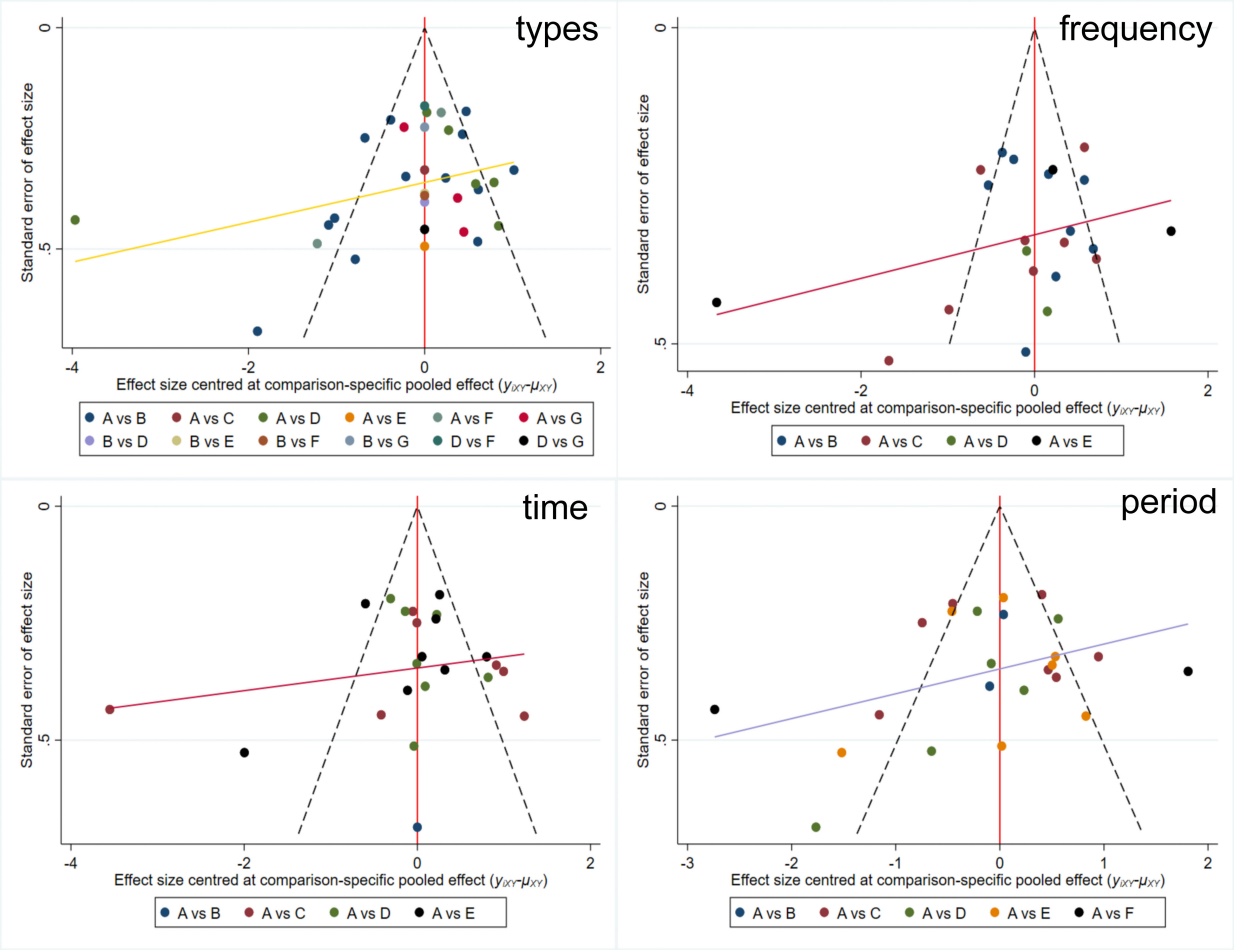
Funnel plot of publication bias.

Publication bias was assessed using Egger’s regression test. The results indicated no statistically significant bias for any analysis (p>0.05). This finding was visually corroborated by funnel plots in **Figure 5**, which showed approximate symmetry for comparisons related to exercise types, frequency, time, and period. Together, the statistical test and visual inspection suggest that publication bias is unlikely to have substantially influenced the meta-analytic results.

## Discussion

This study utilized a network meta-analysis to investigate the effects of exercise type, frequency, time, and period on CLBP in adults. The analysis quantified the effect size for each parameter and established a hierarchical ranking of their efficacy. This provides an evidence based framework to optimize exercise prescription, offering crucial guidance for developing precise rehabilitation protocols. The findings suggest that traditional exercise techniques may be superior to other modalities for improving CLBP. Regarding dosage, the combination with the highest probabilistic ranking comprised sessions lasting 15–20 min, administered 6–7 T/W, over a period of at least 16 weeks. This should not be interpreted as a definitive optimal regimen given clinical heterogeneity, indirect comparisons in NMA, and the non-factorial study design.

The findings indicate that traditional exercise types are an effective intervention, capable of significantly alleviating symptoms in adults with CLBP. While previous research has established the efficacy of Western-originated approaches such as Pilates (Yu et al., 2023), McKenzie therapy (Hayden et al., 2021), and aerobic exercise (Meng and Yue, 2015), this analysis suggests traditional types may offer a distinct advantage. The proposed superiority of these types may be attributed to their unique movement principles. Traditional exercises typically emphasize slow, controlled, and mindful motions, which help modulate muscle tone and reduce the tension-related pain common in CLBP (Lamoth et al., 2006). Furthermore, this modality integrates core stability, postural alignment, muscular coordination, strength, flexibility, and breath regulation, all contributing to symptom improvement (Jansen et al., 2011). The characteristic fluidity and continuity of movement, combined with a focus on synchronized breathing and mental focus, are posited to enhance lumbar proprioception and core stability, providing a multifaceted therapeutic effect (Smith et al., 2022).

The findings indicate that a single session duration of 15–20 min is optimal for improving CLBP. This duration provides sufficient stimulus to promote muscle relaxation, enhance core strength, and improve joint flexibility, while minimizing the risk of muscle fatigue or discomfort associated with longer sessions. Consequently, it more effectively alleviates pain and supports the rehabilitation process (Yu et al., 2023). Furthermore, a frequency of 6–7 T/W demonstrates a significant advantage. However, this high frequency (daily training) may present substantial feasibility and adherence challenges for real-world patients with busy schedules, physical limitations, or low motivation. Strict daily implementation may not be clinically practical or sustainable in routine care. Such frequent, consistent intervention promotes sustained adaptive responses within the musculoskeletal system. Therefore, the statistically highest-ranked frequency should be interpreted cautiously; a more moderate, individually tolerable frequency (such as 3–5 times per week) may be preferable to balance efficacy and long-term adherence in clinical practice. Daily training may accelerate the remodelling of motor patterns through repeated activation of muscular and neural circuits, fostering myelin regeneration and spinal circuit recovery (Wu et al., 2025). Post-exercise, muscle fibres and connective tissues undergo a cycle of micro-damage, repair, and super compensation. A near-daily frequency ensures that each subsequent stimulus is applied while the body is still in a super compensated state, creating a cumulative effect that accelerates gains in strength, endurance, and spinal stability (Gouveia et al., 2022). This frequent training also enhances neural adaptation. Through repetitive proprioceptive input and motor pattern reinforcement, the central nervous system improves its precise recruitment of core musculature, such as the multifidus and transversus abdominis (Li et al., 2022). This refined neuromuscular control helps rapidly reestablish dynamic stability of the lumbar-pelvic region and corrects the aberrant movement patterns associated with chronic pain.

The findings of this study demonstrate that an intervention period exceeding 16 weeks is optimal for improving CLBP. This extended duration is likely necessary because central sensitization, a hallmark of chronic pain, often requires prolonged intervention to reverse (Fernández-Rodríguez et al., 2022). Additionally, CLBP involves persistent pathological adaptations such as muscle imbalance, ligamentous laxity, and intervertebral disc degeneration, which cannot be fully corrected in a short timeframe. A longer intervention period allows sufficient time for physiological adaptation, promoting sustained therapeutic benefits (Li et al., 2021). As the intervention progresses, the body gradually establishes a new, stable equilibrium, resulting in more consistent pain relief and functional recovery (Babiloni-Lopez et al., 2024). Furthermore, long term intervention supports not only physical improvement but also the development of healthier lifestyle and exercise habits, which are crucial for lasting self-management.

### Limitations

This study has several limitations. First, notable clinical and methodological heterogeneity existed across included trials, with differences in population characteristics, baseline pain levels, and research settings, which may reduce the stability of pooled effect estimates.

Second, substantial variation in intervention content and implementation was observed across exercise protocols, which may limit the comparability and reproducibility of the dose−response findings.

Third, several included trials had unclear or high risk of bias in randomization, allocation concealment, or blinding, which may lower the overall certainty of evidence.

Fourth, SUCRA rankings reflect relative probabilistic efficacy rather than definitive clinical superiority, and thus require cautious interpretation to avoid overstatement of between−intervention differences.

Finally, existing literature provides scarce data on exercise intensity, limiting a deeper exploration of dose-response relationships. Subsequent studies should include more trials with standardized intensity metrics to address this gap.

## Conclusion

This study systematically investigated the influence of different exercise parameters on CLBP in adults. Based on probability−based SUCRA rankings, the combination of traditional exercise, 15–20 min per session, 6–7 times per week, and ≥16 weeks of intervention showed the highest probability of favourable effects rather than a definitive optimal regimen. This finding should not be interpreted as a definitively optimal intervention, but rather as a combination that may be associated with better probabilistic efficacy for pain relief in adults with CLBP. This finding should be interpreted as a probabilistic ranking rather than a definitive, universally optimal exercise prescription. The proposed rationale is that this regimen provides moderate stimulation sufficient to enhance core muscle strength and improve joint flexibility, thereby alleviating muscular tension. Simultaneously, its high frequency and extended duration promote sustained adaptation within the musculoskeletal system, fostering a continuous rehabilitation effect.

## Data Availability

The original contributions presented in the study are included in the article/supplementary material, further inquiries can be directed to the corresponding author.
